# Walking strategies in subjects with congenital or early onset strabismus

**DOI:** 10.3389/fnhum.2014.00484

**Published:** 2014-07-10

**Authors:** Irene Aprile, Maurizio Ferrarin, Luca Padua, Enrica Di Sipio, Chiara Simbolotti, Sergio Petroni, Costanza Tredici, Anna Dickmann

**Affiliations:** ^1^SM Provvidenza Movement Laboratory, Don Carlo Gnocchi Foundation, IRCCSMilan, Rome, Italy; ^2^Biomedical Technology Department, Don Carlo Gnocchi Foundation, IRCCSMilan, Italy; ^3^Neuroscience Department of Catholic UniversityRome, Italy; ^4^Ophthalmology Department, Bambino Gesù Children's HospitalRome, Italy; ^5^Department of Surgical Sciences of Head and Neck, Institute of Ophtalmology, Catholic UniversityRome, Italy

**Keywords:** gait analysis, strabismus, binocular visual field, walking strategy

## Abstract

**Introduction:** In congenital strabismus, sensory adaptations occur hampering the correct development of normal binocular vision. The aim of this study is to investigate if patients with congenital or early onset exotropic or esotropic strabismus adopt different walking strategies with respect to healthy subjects. Our hypothesis is that the abnormal binocular cooperation, occurring in patients with exotropic or esotropic strabismus, could influence neurosensorial adaptation of the gait pattern.

**Materials and Methods:** Twenty-five patients were enrolled: 19 with esotropic (ESO) and 6 with exotropic strabismus (EXO). All patients underwent an ophthalmological and orthoptic evaluation. Biomechanical data were collected using a stereophotogrammetric system and a force platform. Twenty-seven age-matched healthy subjects (HS) were used as controls.

**Results:** The comparison between patients with ESO and patients with EXO strabismus showed that the maximal power at the knee and at the ankle was lower in EXO group (*p* < 0.01 and *p* < 0.05, respectively). The step width was statistically different between ESO and EXO groups (*p* < 0.01), lower in patients with ESO and higher in patients with EXO strabismus when compared with HS (though not statistically significant). The deviation angle values showed a relationship with the step width (at the near fixation *p* < 0.05) and with the maximal power at the knee and at the ankle (at the far fixation for the knee *p* < 0.001 and for the ankle *p* < 0.05; at the near fixation for the knee *p* < 0.05): in the patients with EXO the increased angle deviation is related to larger step width and to lower power at the knee and at the ankle. In the patients with ESO strabismus this relationship is less robust.

**Discussion:** Patients with EXO and ESO strabismus adopt different strategies to compensate their walking difficulties, and these strategies are likely due to an expanded binocular visual field in patients with EXO and to a reduced visual field in patients with ESO strabismus.

## Introduction

Strabismus is a condition characterized by a misalignment of the visual axes which leads to anomalies of the sensory and motor balance of both eyes. Depending on the age at onset, strabismus may determine different consequences: if it is congenital or starts in the first months after birth (that is during the period of maximal cortical plasticity) sensory adaptations (suppression, anomalous retinal correspondence) occur hampering the correct development of normal binocular vision and stereopsis (normal binocular cooperation). When strabismus occurs in the period in which cortical plasticity is no longer possible, sensory adaptations do not take place and patients will experience diplopia and confusion.

Strabismus may be classified according to various clinical aspects, i.e., age at onset, comitant or non-comitant (if the amount of deviation is the same in all gaze positions or not), alternating (if the patient can freely fix with each eye), or monocular (if the same eye is always the fixing one). When strabismus is always present, it is called heterotropia, if it is latent it is called heterophoria.

**Figure 1:**
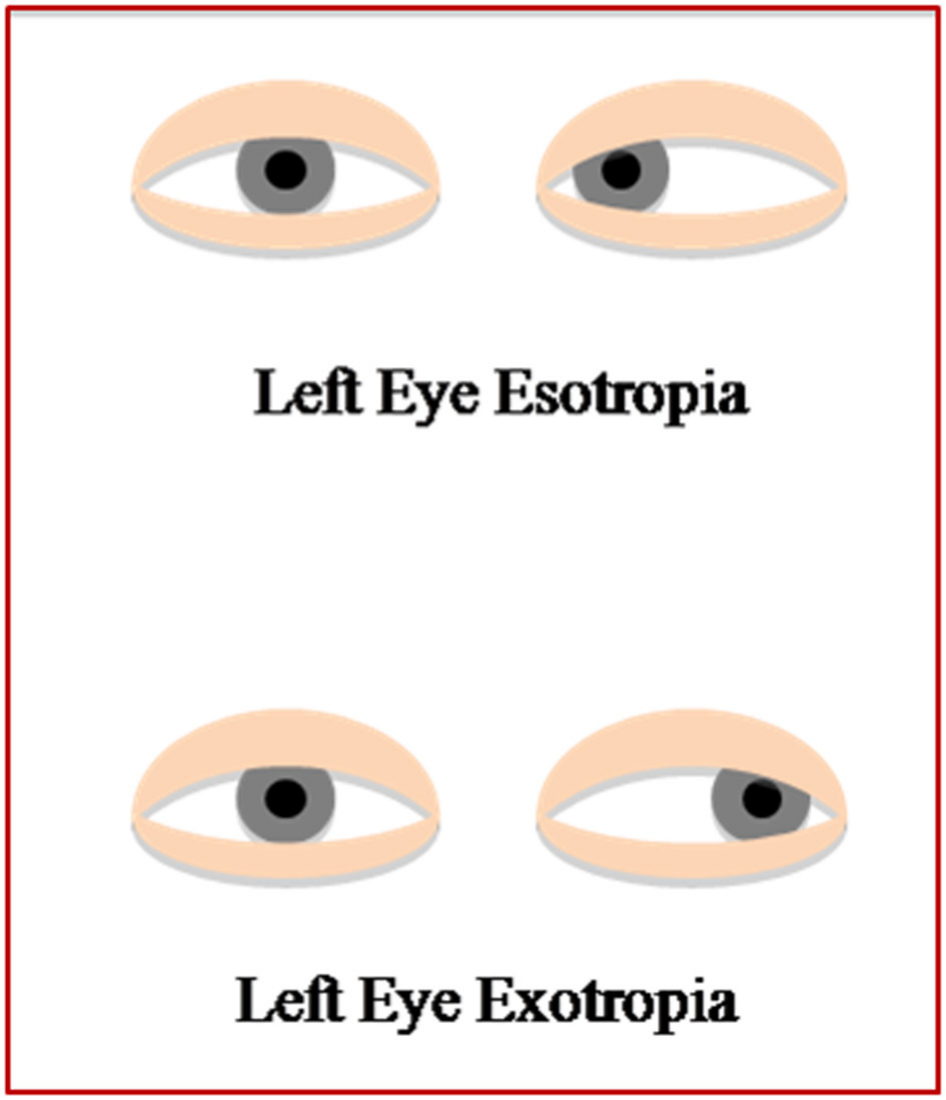
**Description of the eye direction, deviated toward the temple in patients with EXO and deviated toward the nose in patients with ESO**.

Direction of deviation is a common manner to describe strabismus: if the direction of the deviated eye is toward the temple it is an exodeviation, whereas, if the direction of deviation is toward the nose it is an esodeviation (Figure 1). As a rule, the amount of the deviation (generally measured in Prismatic Diopters - PD) is negative for exodeviation and positive for esodeviation.

Walking is a complex task for the peripheral and central nervous system, which has to generate and control dynamic instability in order to produce the propulsive force needed for forward progression. Walking effectively through a complex environment requires successful integration of both sensory and motor functions. Visual function abnormalities may pose significant challenges to an individual in terms of such integration.

The visual system provides information not only on target distance and presence of obstacles, but also on maintaining balance during walking (Hallemans et al., 2010) adjusting trajectories when an obstacle appears or the target is shifted (Reynolds and Day, 2005).

Vision controls dynamic stability providing information about the environment (objects near and far) thus helping regulate walking both at local (step by step basis) and global (route planning) levels. So, individuals with vision abnormalities may take longer to walk safely through the environment.

A study investigated the effects of optic flow field alterations on spatio-temporal gait parameters and on joint kinematics (Konczak, 1994), and others have studied gait in patients with visual deprivation (Hallemans and Aerts, 2009; Hallemans et al., 2009a,b). In one study a significant influence of reversible visual deprivation on gait parameters has been observed in HS (Iosa et al., 2012).

No studies on gait strategies of subjects with abnormal sensory and motor eyes cooperation, at birth or started in the first months after birth (as in congenital/early onset strabismus), have been reported. Subjects with congenital or early onset strabismus have an abnormal binocular vision since the time of maximal cortical plasticity, and they could develop different walking adaptive strategies with respect to those observed after reversible visual deprivation in HS.

Several authors (Odenrick et al., 1984; Matsuo et al., 2006, 2010; Matheron et al., 2007; Legrand et al., 2011; Gaertner et al., 2013a; Lions et al., 2013; Przekoracka-Krawczyk et al., 2014) found that subjects with congenital or early onset strabismus have a significantly lower static and dynamic balance than HS. However, a binocular visual stimulation plays an important role in postural control of patients with strabismus (Gaertner et al., 2013b).

Our hypothesis is that the abnormal binocular cooperation, occurring in patients with congenital or early onset exotropic or esotropic strabismus, could influence neurosensorial adaptations of the gait pattern.

The aim of this study was to investigate if patients with exotropic or esotropic congenital or early onset strabismus adopt different walking strategies with respect to HS.

## Materials and methods

### Population

A sample of patients with strabismus and a group of age-matched HS were analyzed. Twenty-five patients with strabismus were enrolled in this study: 13 males and 12 females, range 5–50 years. In particular, 6 patients with exotropic strabismus (EXO) of mean age 17.33 years (*SD*: 17.31), and 19 patients with esotropic strabismus (ESO) of mean age 13.16 years (*SD*: 6.64). Inclusion criteria were congenital or early onset strabismus (within 1 year of age) and age ≥ 5 years. All patients were able to walk independently without assistance or walking aids. Exclusion criteria included strabismus acquired after 1 year of age, lack of cooperation for age or mental retardation, presence of systemic or neurological diseases as well as of orthopedic or postural problems. We excluded patients with cognitive impairment, cardiologic diseases that made walking risky, or other diseases liable to cause motor gait impairment (i.e., radiculopathy, bone fracture, etc.). The HS group consisted of 27 subjects (14 females and 13 males, mean age 17.93 years, range 5–46).

All the participants gave their informed consent prior to participating in the study, which complied with the Helsinki Declaration.

### Ophthalmological and orthoptic evaluation

Both patients and HS underwent a complete ophthalmological and orthoptic evaluation. Subjects with strabismus were divided, according to the direction of deviation, into two groups: patients with esotropic and patients with exotropic strabismus (Table 1). In no patient a vertical or cyclotorsional component was associated with the horizontal deviation. The amount of strabismus was always measured by means of Prismatic Cover test, both at far fixation (6 m) and at near fixation (0.40 m), considering that difference in the amount of deviation may depend on the distance of fixation. Due to the presence of a congenital or early onset strabismus a normal binocular vision was absent in all patients.

**Table 1:** **Anthropometric and ophthalmological/orthoptic evaluation of patients with strabismus**.

**PATIENT**	**SEX**	**AGE (yy)**	**Mass (Kg)**	**Stature (m)**	**Visual acuity RE**	**Visual acuity LE**	**Deviation angle (PD) at far fixation**	**Deviation angle (PD) at near fixation**
IMAL	M	5	18	1.10	6/6	6/7.5	XT50	XT′45
PESI	M	5	17	1.10	6/7.5	6/6	XT14	XT′20
MOAD	F	6	21	1.20	6/6	6/7.5	ET10	ET′18
GAFR	M	7	32	1.30	6/12	6/7.5	ET25	ET′30
FASO	F	8	24	1.25	6 / 9.5	6/9.5	ET12	ET′35
INDE	F	7	19	1.21	6/15	6/9.5	ET30	ET′35
CAFI	M	7	24	1.23	6/7.5	6/6	ET35	ET′40
VEAR	M	8	35	1.38	6/7.5	6/9.5	ET6	ET′10
CHVA	F	8	34	1.30	6/6	6/9.5	ET20	ET′18
ALMA	M	8	30	1.32	6/6	6/12	ET75	ET′70
QUTO	M	9	27	1.30	6/7.5	6/6	ET30	ET′35
BOVI	F	10	33	1.40	6/6	6/6	XT2	XT′8
TAIL	F	11	35	1.44	6/9.5	6 / 7.5	ET40	ET′75
DILE	M	11	55	1.55	6/6	6/6	XT8	XT′16
CAMA	F	12	30	1.42	6/6	6/9.5	ET25	ET′35
DESI	F	13	47	1.58	6/9.5	6/12	ET18	ET′20
VARO	M	15	65	1.70	6/6	6/7.5	ET16	ET′20
CHAD	M	16	73	1.80	6/6	6/6	ET35	ET′30
SULA	F	19	40	1.58	6/9.5	6/7.5	ET14	ET′8
FEAN	M	23	80	1.76	6/6	6/7.5	XT16	XT′40
COAR	F	23	49	1.63	6/12	6/7.5	ET50	ET′60
PAIL	F	24	72	1.81	6/6	6/6	ET4	ET′8
PADA	M	24	75	1.80	6/7.5	6/6	ET12	ET′8
NALA	F	24	55	1.53	6/6	6/6	ET20	ET′14
CAFR	M	50	76	1.74	6/6	6/6	XT35	XT′35

RE, right eye; LE, left eye; PD, Prismatic Diopters; ET, esotropia at far fixation; ET ', esotropia at near fixation; XT, exotropia at far fixation; XT′, exotropia at near fixation.

In the HS group all subjects were emmetropic or with a best corrected visual acuity of 6/6 and showed a normal binocular vision and stereopsis ≥60″ at the orthoptic evaluation.

#### Gait analysis

The gait analysis was performed using the Smart D500 stereo-photogrammetric systems (BTS Bioengineering, Milan, Italy). The system consists of eight infrared cameras (sampling rate of 200 Hz) to acquire movement of reflective spherical markers placed on anatomical landmarks. Kinetic data were acquired at 1 kHz using a single 600 × 400 mm piezoelectric force platform (Kistler, Winterthur, Switzerland). The patients were equipped with 22 retro-reflective markers, according to Davis protocol (Davis et al., 1991). The marker-set is composed of 18 markers directly applied to the skin and 4 wands placed at 1/3 the length of the body segment. In particular, it places a wand on the femur and on the leg, so that the plane containing the three points is parallel to the frontal plane. For each subject anthropometric measures were collected; to increase the reliability of the biomechanical measures, markers placement and anthropometric measures were performed by the same operator (Winter, 1979).

Before formal measurements were started, practice sessions were performed to let participants become familiar with the procedure. Both patients and HS were asked to walk barefoot straight ahead along a level surface of approximately 6 m at their self-selected speed. Ten linear walking trials were acquired for each subject. To avoid fatigue, groups of 5 trials were separated by 1 min rest.

Three-dimensional (3D) kinematics, dynamics were considered if complete data of at least one stride (indifferently right or left) were available, otherwise the trial was discarded. Trials, which presented evident artifacts due to technical problems (missing detection of some markers or improper foot-strike on platform), were excluded.

#### Data analysis

Three-dimensional marker trajectories were tracked using a frame-by-frame tracking system (Smart Tracker-BTS, Milan, Italy). Data were processed using 3D reconstruction software (SMARTAnalyzer, BTS, Milan, Italy) and MATLAB software (MATLAB 7.4.0, MathWorks, Natick, MA, USA).

The following parameters were considered:

*Spatio-temporal parameters.* Gait cycle duration was defined as the interval between two consecutive heel contacts of the same foot. To reduce the possible variability due to the age-range of the sample, we have normalized all height-dependent gait parameters (i.e., step length, step width and walking speed) by subject height. The following parameters were calculated: cycle time [s], stance [%], swing [%] and double support phase duration [%], step length [%H], step width [%H], cadence [step/min], walking speed [%H/s]. All dynamic parameters were normalized to body weight.
*Kinematic parameters.* The lower limb joint kinematics in the sagittal plane was considered and the hip, knee and ankle joint range of motion (ROM) [°] were calculated.
*Kinetic parameters.* The maximal power and maximal moment in early and late stance at the hip and knee were calculated; for the ankle the maximal power and maximal moment in late stance were considered. Early stance is defined as the percentage of the gait cycle ranging from 0 to 30, and late stance is defined as the percentage of the gait cycle ranging from 30 to 60.

#### Statistical analysis

The statistical analysis was performed using the STATSOFT (Tulsa, OK, USA) package. All data were tested for normality with the Shapiro–Wilk test. Since the variables were not normally distributed, the Mann-Whitney test was used for all the investigated variables (spatio-temporal, kinematic and kinetic data) to determine differences between the following group pairs: patients with strabismus vs. HS, patients with ESO vs. patients with EXO strabismus, patients with ESO strabismus vs. HS, patients with EXO strabismus vs. HS. Moreover, the Spearman's rank correlation coefficient test was used to evaluate the correlations between deviation angle values at far and near fixation and spatio-temporal, kinematic and kinetic data, was used. The significance level was set for all parameters at *p* < 0.05.

## Results

### Comparison between subjects with strabismus and healthy subjects

In Table 2 mean, standard deviation, and *p*-value of age, anthropometric measures and gait analysis results are reported. No significant differences were found in age, stature, and mass between patients and HS. Kinetic results showed a significantly lower maximal moment in patients than in HS at the hip (*p* < 0.001 in early stance, and *p* < 0.0001 in late stance), at the knee (*p* < 0.0001 in early and late stance) and at the ankle (*p* < 0.05). Moreover a significantly lower range of motion of the knee was found in patients than in HS (*p* < 0.01).

**Table 2:** **Spatio-temporal, kinematic, and kinetic parameters in patients and in HS**.

**Variable**	**Patients (25 cases)**	**Healthy Subjects (27 cases)**	***p-*value**
	**Mean (*SD*)**	**Mean (*SD*)**	
Age [yy]	14.04 (9.98)	17.93 (12.20)	NS
**ANTHROPOMETRIC**			
Mass [Kg]	42.64 (20.57)	46.85 (20.25)	NS
Stature [m]	1.46 (0.23)	1.48 (0.25)	NS
**TIME DISTANCE**			
Cycle Time [s]	1.04 (0.13)	1.01 (0.15)	NS
Stance [%]	59.32 (1.65)	58.87 (1.75)	NS
Swing [%]	40.96 (1.66)	41.13 (1.80)	NS
DBS [%]	8.71 (1.32)	8.53 (1.72)	NS
Step length [%H]	34.50 (2.60)	34.54 (2.09)	NS
Step width [%H]	9.49 (1.91)	9.52 (1.63)	NS
Cadence [step/min]	117.78 (15.47)	122.01 (20.27)	NS
Walking speed [%H/s]	73.38 (12.63)	75.64 (14.97)	NS
**KINEMATIC**			
Hip SAG ROM [°]	46.10 (5.19)	46.11 (3.51)	NS
Knee SAG ROM [°]	57.59 (4.78)	60.71 (3.93)	<0.01
Ankle SAG ROM [°]	28.43 (5.33)	30.11 (5.52)	NS
**KINETIC**			
Hip max moment (early stance) [N*m/Kg]	0.70 (0.10)	0.79 (0.06)	<0.001
Hip max moment (late stance) [N*m/Kg]	0.19 (0.10)	0.06 (0.05)	<0.0001
Knee max moment (early stance) [N*m/Kg]	0.30 (0.09)	0.41 (0.06)	<0.0001
Knee max moment (late stance) [N*m/Kg]	0.10 (0.04)	0.13 (0.03)	<0.05
Ankle max moment [N*m/Kg]	1.30 (0.17)	1.46 (0.08)	<0.0001
Hip max power (early stance) [W/Kg]	1.19 (0.37)	1.29 (0.21)	NS
Hip max power (late stance) [W/Kg]	0.69 (0.21)	0.59 (0.15)	NS
Knee max power (early stance) [W/Kg]	0.46 (0.16)	0.49 (0.13)	NS
Knee max power (late stance) [W/Kg]	0.43 (0.15)	0.46 (0.18)	NS
Ankle max power [W/Kg]	3.07 (0.69)	3.08 (0.56)	NS

### Comparison between esotropic and exotropic subjects and healthy subjects

In Table 3, age, anthropometric and gait analysis data (mean, *SD* and *p*-values) in ESO, EXO and HS are reported. Furthermore, the comparison between each groups of patients and HS (ESO vs. HS, EXO vs. HS) and the comparison between the two groups of patients (ESO vs. EXO) are shown. No differences in age, stature and mass were found among the three groups.

**Table 3:** **Comparison between groups: patients with esotropic strabismus (ESO) vs. Healthy Subjects (HS); patients with exotropic strabismus (EXO) vs. Healthy Subjects (HS)**.

**Variable**	**Patients with ESO**	***p*-value**	**Patients with EXO**	***p-*value**	***p-*value**
	**Mean (*SD*)**	**(ESO vs. HS)**	**Mean (*SD*)**	**(EXO vs. HS)**	**(ESO vs. EXO)**
Age [yy]	13.00 (6.69)	NS	17.33 (17.31)	NS	NS
t**ANTHROPOMETRIC**					
Mass [Kg]	41.42 (18.43)	NS	46.50 (28.03)	NS	NS
Stature [m]	1.46 (0.21)	NS	1.44 (0.30)	NS	NS
t**TIME DISTANCE**					
Cycle Time [s]	1.04 (0.13)	NS	1.04 (0.14)	NS	NS
Stance [%]	59.28 (1.63)	NS	59.44 (1.89)	NS	NS
Swing [%]	41.09 (1.62)	NS	40.56 (1.89)	NS	NS
DBS [%]	8.66 (1.19)	NS	8.86 (1.79)	NS	NS
Step Length [%H]	35.11 (2.36)	NS	32.58 (2.55)	NS	NS
Step Width [%H]	8.86 (1.16)	NS	11.51 (2.52)	NS	<0.01
Cadence [step/min]	117.86 (15.45)	NS	117.53 (17.00)	NS	NS
Walking speed [%H/s]	74.68 (11.73)	NS	69.27 (15.60)	NS	NS
t**KINEMATIC**					
Hip SAG ROM [°]	47.23 (5.09)	NS	42.68 (4.11)	NS	NS
Knee SAG ROM [°]	58.59 (4.13)	<0.05	54.40 (5.70)	<0.05	NS
Ankle SAG ROM [°]	29.63 (5.12)	NS	24.64 (4.42)	NS	NS
t**KINETIC**					
Hip max moment (early stance) [N*m/Kg]	0.70 (0.08)	<0.0001	0.71 (0.17)	NS	NS
Hip max moment (late stance) [N*m/Kg]	0.15 (0.09)	<0.0001	0.29 (0.06)	<0.0001	<0.01
Knee max moment (early stance) [N*m/Kg]	0.31 (0.09)	<0.001	0.24 (0.06)	<0.0001	NS
Knee max moment (late stance) [N*m/Kg]	0.10 (0.03)	NS	0.09 (0.05)	NS	NS
Ankle max moment [N*m/Kg]	1.33 (0.13)	<0.0001	1.21 (0.25)	<0.01	NS
Hip max power (early stance) [W/Kg]	1.19 (0.36)	NS	1.21 (0.44)	NS	NS
Hip max power (late stance) [W/Kg]	0.70 (0.24)	NS	0.64 (0.09)	NS	NS
Knee max power (early stance) [W/Kg]	0.51 (0.15)	NS	0.39 (0.07)	<0.001	<0.01
Knee max power (late stance) [W/Kg]	0.43 (0.16)	NS	0.41 (0.08)	NS	NS
Ankle max power [W/Kg]	3.25 (0.59)	NS	2.49 (0.69)	NS	<0.05

**Figure 2:**
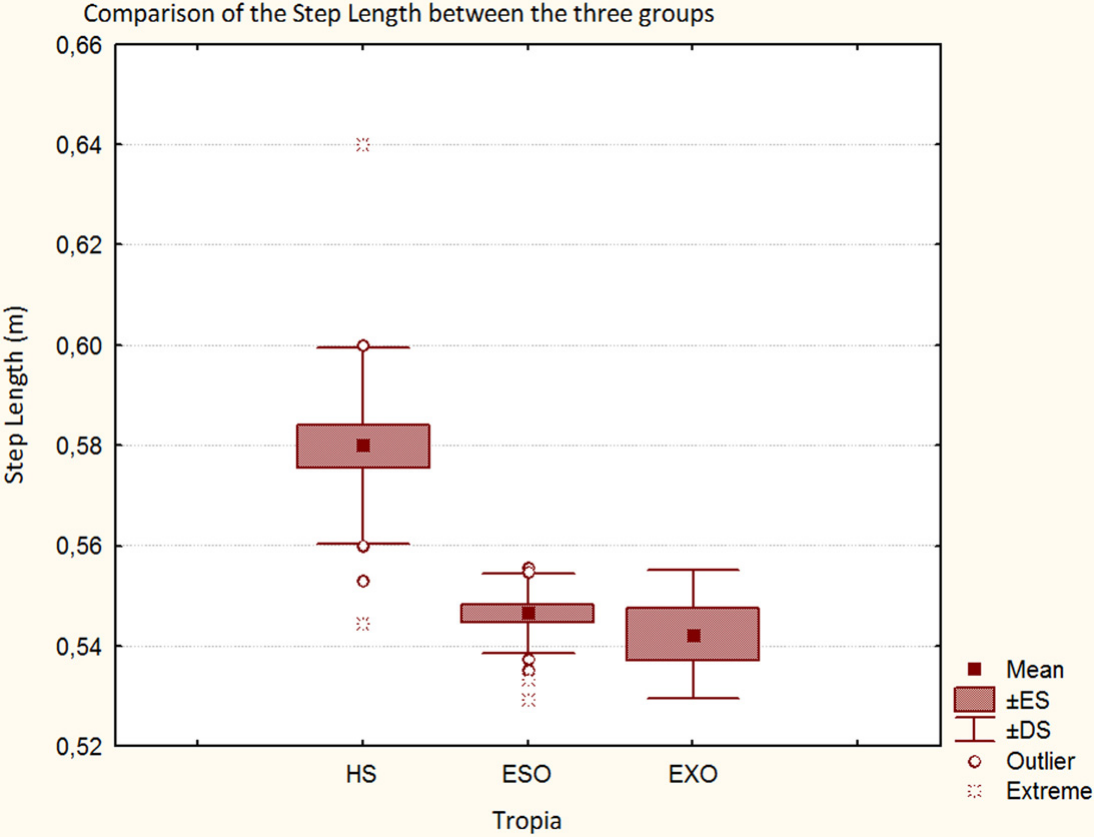
**Step length in HS, in patients with ESO and in patients with EXO**.

**Figure 3:**
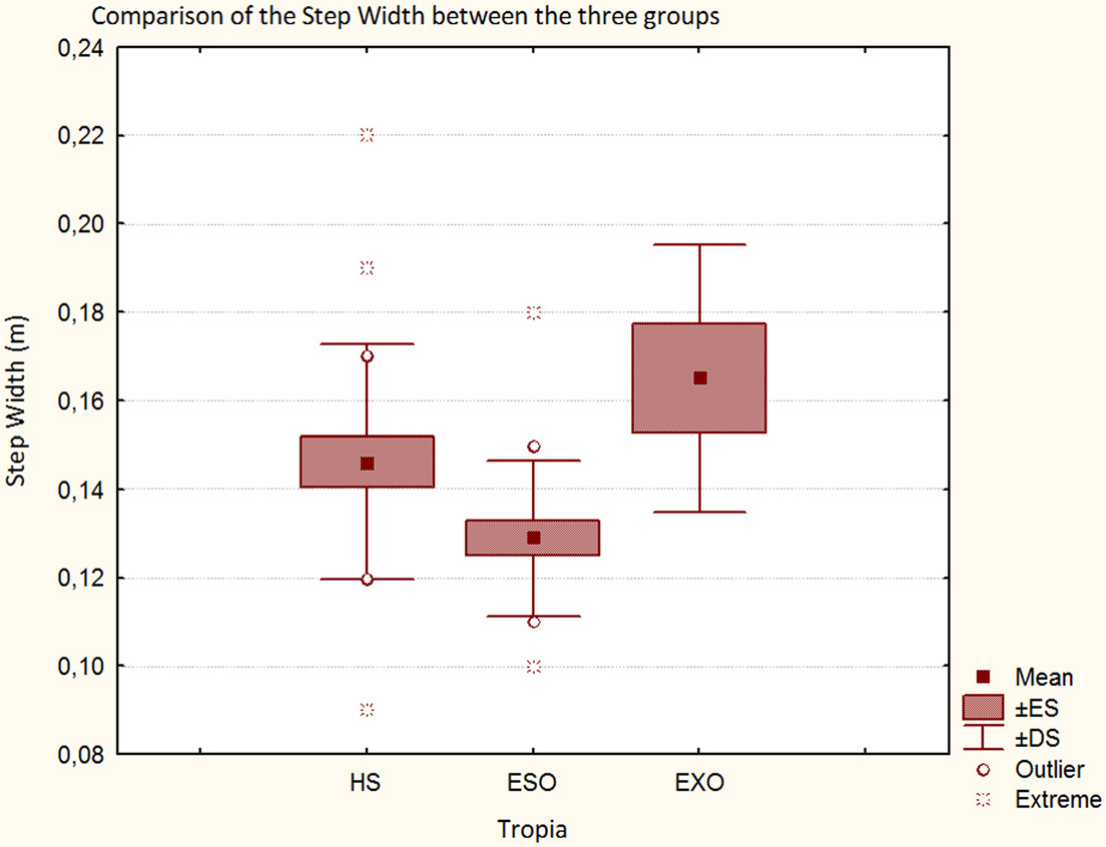
**Step width in HS, in patients with ESO and in patients with EXO**.

Regarding spatio-temporal results, Figure 2 describes as the step length progressively reduced in the three groups (HS, ESO, and EXO). The step width was smaller in patients with ESO and wider in patients with EXO when compared with HS, respectively (Figure 3). A significant difference in the step width was observed when patients with ESO and patients with EXO were compared (*p* < 0.01, see Table 3).

Regarding kinematic results patients with ESO and patients with EXO had a significantly reduced ROM of the knee than HS (*p* < 0.05). A reduction (though not statistically significant) of the hip ROM was found between patients with EXO and HS and of the hip and ankle ROM comparing ESO and EXO groups.

**Figure 4:**
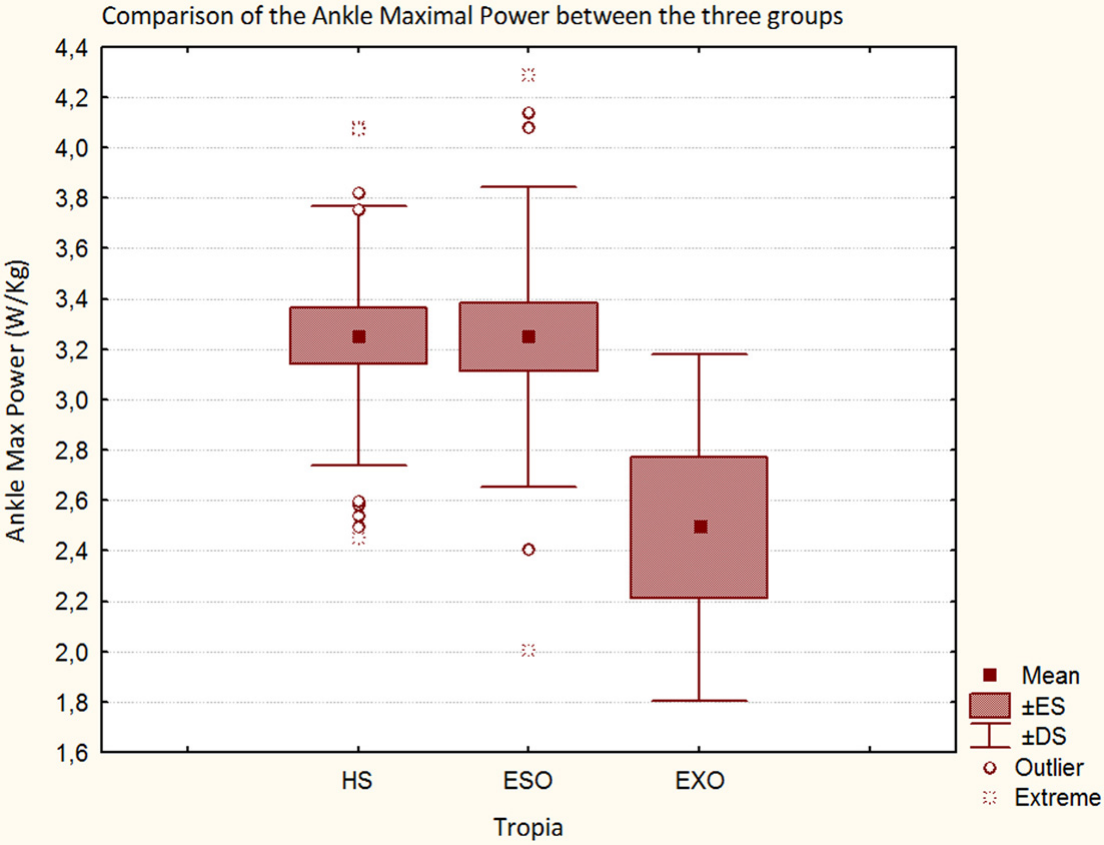
**Maximal Power at the ankle in HS, in patients with ESO and in patients with EXO**.

Regarding kinetic results, comparing patients with ESO and HS significant differences in hip moment (in early and late stance, *p* < 0.001), in knee moment (in early stance, *p* < 0.001), and in ankle moment were found (*p* < 0.0001), (see Table 3). Comparing patients with EXO and HS, significant differences in hip moment (in late stance *p* < 0.0001), in knee moment (in early stance *p* < 0.0001), in ankle moment (*p* < 0.01) were found. Moreover a significant reduced maximal power at the knee (in early stance) was found between patients with EXO and HS (*p* < 0.001). Finally, comparing patients with ESO and patients with EXO, significant differences in hip moment (in late stance, *p* < 0.01), and mainly in the maximal power at the knee (in early stance, *p* < 0.01) and at the ankle (*p* < 0.05) were found. The maximal power at the knee and at the ankle is lower in patients with EXO than patients with ESO. Moreover, the maximal power at the ankle is similar between HS and patients with ESO but strongly reduced in the patients with EXO (Figure 4).

### Relationship between orthoptic and gait parameters

**Figure 5:**
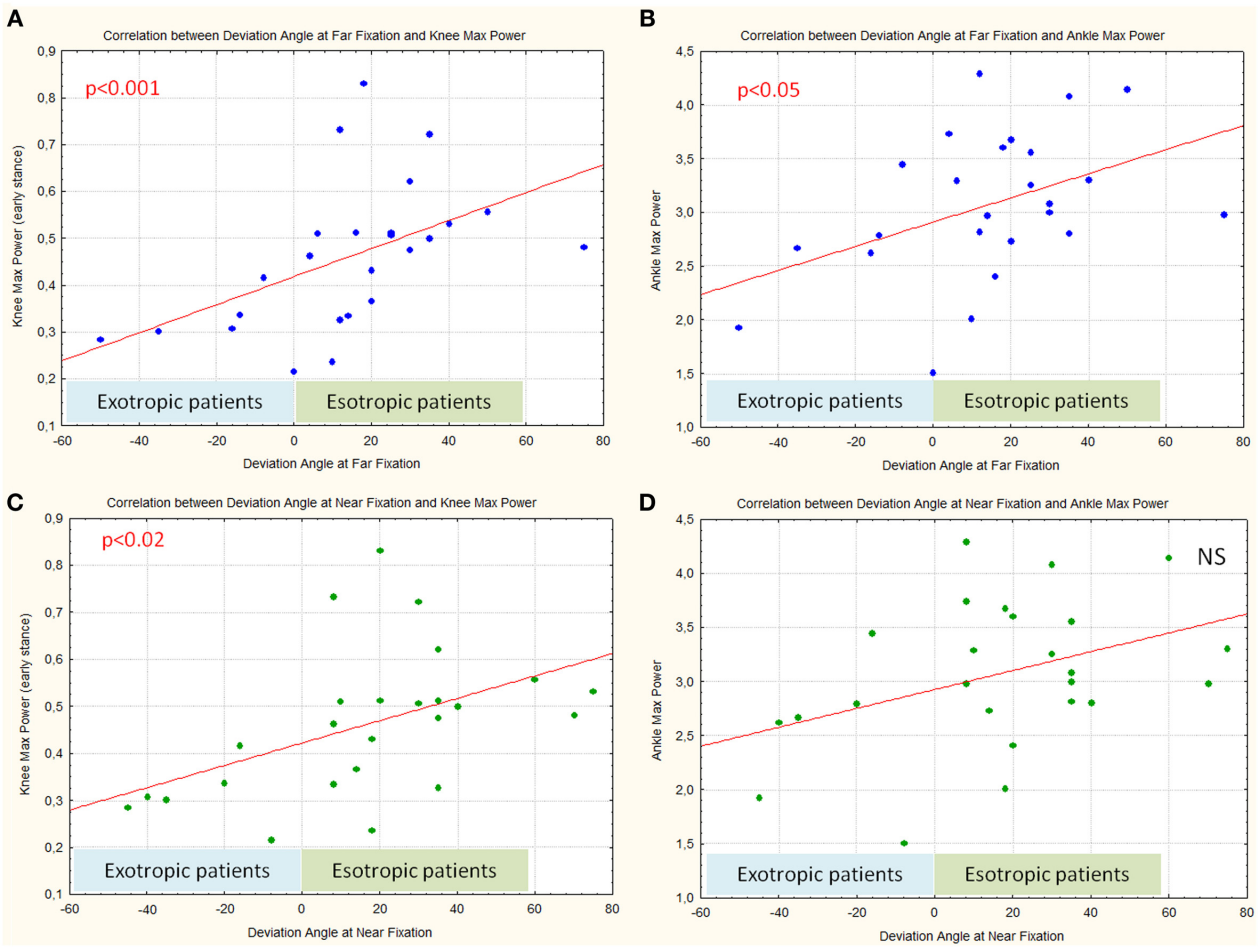
**Correlation between deviation angle values at far (A,B) and near fixation (C,D) and Maximal Power at the knee and at the ankle**.

The deviation angle values at far fixation showed a statistically significant relationship with the maximal power at the knee (*p* < 0.001) and at the ankle (*p* < 0.05): in patients with more negative values (expression of the severity of the motor misalignment in subjects with exotropic strabismus) a lower power at the knee and at the ankle was observed (Figures 5A,B). The deviation angle values at near fixation showed a significant correlation with the maximal power at the knee (*p* < 0.05): in patients with more negative values a lower power at the knee (Figure 5C) was observed. Regarding the maximal power at the ankle, a similar relationship was observed but without statistical significance (Figure 5D).

**Figure 6:**
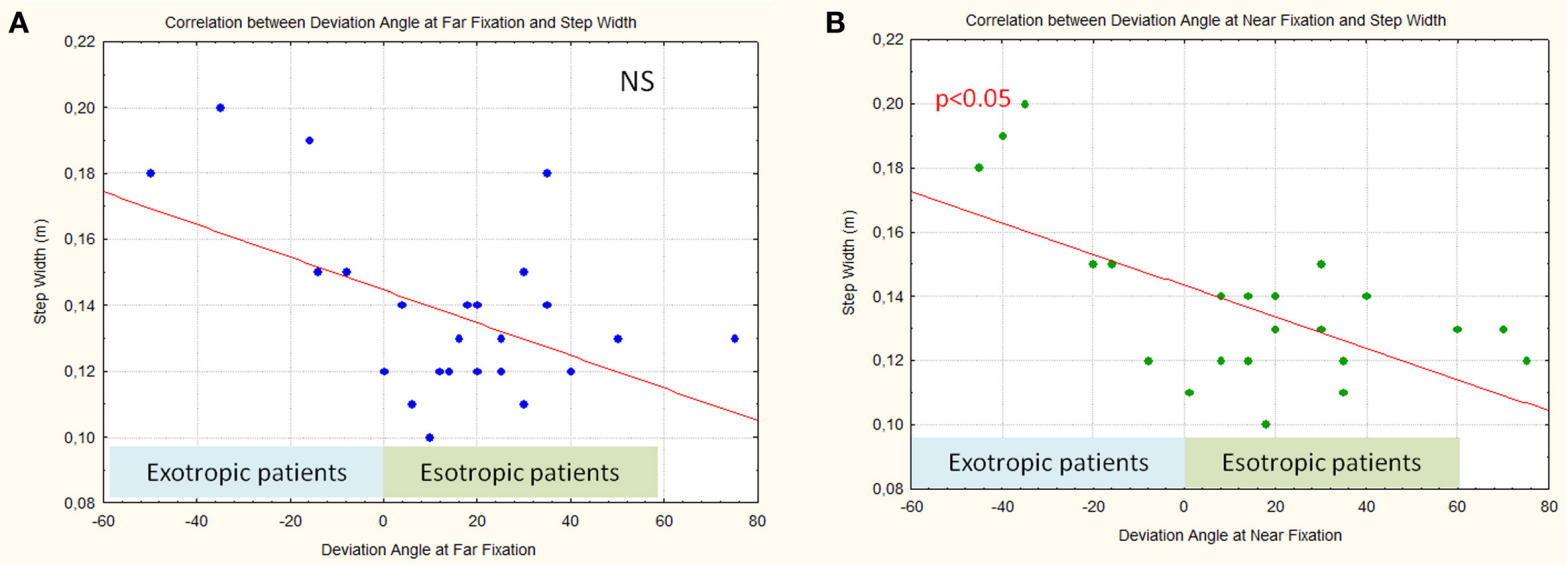
**Correlation between deviation angle values at far fixation (A) and near fixation (B) and step width**.

Furthermore an interesting significant relation was observed between the step width and angle values at near fixation (*p* < 0.05): patients with EXO with more negative values had a larger step width (Figure 6A), moving toward more positive values; patients with ESO had a decreased step width. A similar relationship, between the step width and angle values at far fixation was observed (though not statistically significant).

It is interesting to underline that an accurate analysis of Figures 5, 6 shows that, considering only patients with ESO, the correlation between deviation angle and gait parameters (step width and power at the knee and ankle) seems less robust. In fact, when patients with EXO and patients with ESO were separately considered we found that patients with EXO maintain a significant relationship between step width and power at knee with deviation angle (*p* < 0.05), while patients with ESO maintain a significant relationship only between step width and deviation angle (*p* < 0.05).

## Discussion

The results of our study show the presence of different walking strategies in patients with ESO and EXO strabismus. In particular we observed significant differences in step width between the two groups. It is important to note that the larger step width is generally considered as a feature characterizing walking instability, while a smaller step width is recognized as an expression of good stability (Schrager et al., 2008). Regarding this parameter, patients with ESO and EXO showed different behavior when compared to HS: patients with ESO had a smaller step width than HS while patients with EXO had a larger step width than HS.

**Figure 7:**
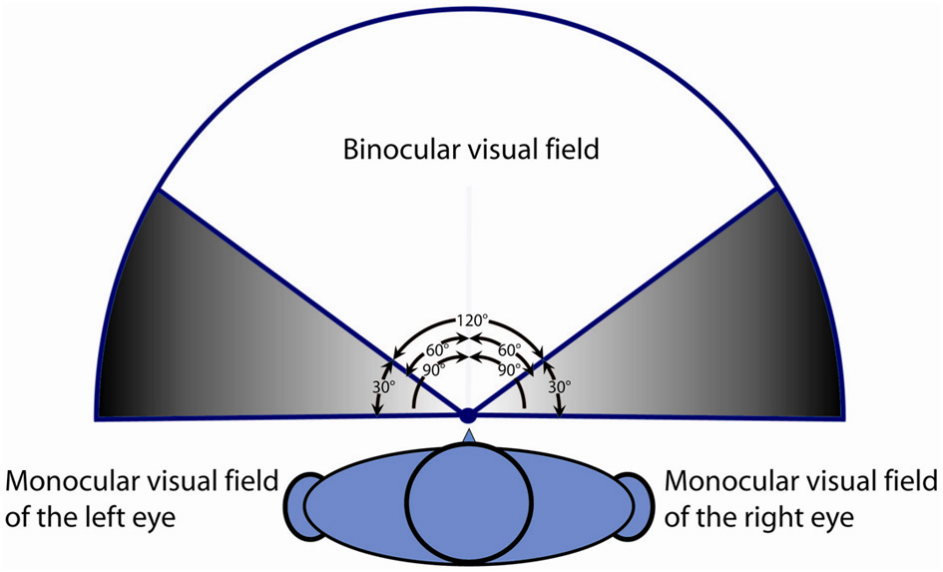
**Description of the normal binocular visual field and monocular visual field of the right and left eye**.

A possible explanation might rely on the characteristics of Binocular Visual Field (BVF) in patients with strabismus. The integrity of binocular visual field is recognized as an important factor influencing walking (Graci et al., 2009, 2010). In normal subjects the visual fields of both eyes widely overlap in the central part of each hemi-field (binocular visual field), as only a small part (the “temporal crescent” emerging from the more medial part of the nasal retina) is seen monocularly (Figure 7).

In patients with strabismus, despite a normality of visual fields when monocularly tested, BVF shows some anomalies concerning its extent (also depending on the direction of deviation eso/exo) when compared to HS. In esotropia BVF is more restricted and, in many cases, can show an expansion after strabismus surgery (Wortham and Greenwald, 1989; Kushner, 1994; Rosenbaum, 1999). Conversely, patients with EXO may exhibit an expanded visual field (von Noorden and Campos, 2002), in some way similar to the vision experienced by animals characterized by lateralization of the eyes (in this condition binocular overlap of visual field is absent or extremely limited). Especially in subjects with EXO, who can easily alternate eye fixation, the brain receives images at each moment from one eye or the other, suppressing the visual field originated by the non-fixing eye, and this could negatively influence the gait. Probably this behavior in patients with EXO could explain the larger step width and the worse walking performance with less power at the knee and ankle than patients with ESO.

Our results show a significant correlation between step width and near deviation angle value. In patients with EXO the step width proportionally increased with the angle of deviation. This is in agreement with the fact that modifications in perception of BVF seem to strongly depend on the amount of ocular misalignment. A similar correlation trend was observed between step width and the amount of deviation at far fixation (though without statistical significance). The small number of patients with EXO enrolled in the study could explain this result. The presence of a reduced BVF in patients with ESO and an expanded one in patients with EXO might lead to a gait adjustment based on modifications of step width. The direct correlation between the amount of strabismus and step width could support the hypothesis of an adaptation strategy used to compensate the BVF anomalies.

Kinetic data showed that in patients with EXO power at the ankle, and above all at the knee joints, proportionally reduces when the angle of deviation increases. This supports the hypothesis of a decrease of gait performance in EXO patients proportional to the angle of deviation, which induce a more cautious gait characterized by enlarged step width and reduced maximum power at knee and ankle joint level.

It is interesting to note that when patients with ESO and patients with EXO were separately considered, the correlation between step width and deviation angle was maintained in both groups, the correlation between knee power and deviation angle was lost in patients with ESO. To confirm this finding, a larger sample of patients is needed.

The results of our study show that subjects with EXO and ESO adopt different strategies to compensate their walking difficulties and these different strategies are probably due to an expanded visual field in patients with EXO and in a reduced visual field in patients with ESO.

Further studies should also be addressed to which other aspects of the binocular vision disorders can influence the different walking strategies that patients with ESO and EXO adopt. Moreover, a short and long follow-up could be useful to verify the natural evolution of these different strategies in the two groups of patients.

Our results about walking strategies of patients with congenital or early onset ESO and EXO, suggest the application of integrated rehabilitation therapies focused not only on gait training but also on visual field training.

### Conflict of interest statement

The authors declare that the research was conducted in the absence of any commercial or financial relationships that could be construed as a potential conflict of interest.

## References

[B1] Davis R. B. 3rd. Ounpuu S. Tyburski D. Gage J. R. (1991). A gait data collection and reduction technique. Hum. Mov. Sci. 10, 575–587 10.1016/0167-9457(91)90046-Z

[B2] Gaertner C. Creux C. Espinasse-Berrod M. A. Orssaud C. Dufier J. L. Kapoula Z. (2013a). Postural control in nonamblyopic children with early-onset strabismus. Invest. Ophthalmol. Vis. Sci. 54, 529–536 10.1167/iovs.12-10586 23150621

[B3] Gaertner C. Creux C. Espinasse-Berrod M. A. Orssaud C. Dufier J. L. Kapoula Z. (2013b). Benefit of bi-ocular visual stimulation for postural control in children with strabismus. PLoS ONE 8:e60341 10.1371/journal.pone.0060341 23565228 PMC3614554

[B4] Graci V. Elliott D. B. Buckley J. G. (2009). Peripheral visual cues affect minimum-foot-clearance during overground locomotion. Gait Posture 30, 370–374 10.1016/j.gaitpost.2009.06.011 19628392

[B5] Graci V. Elliott D. B. Buckley J. G. (2010). Utility of peripheral visual cues in planning and controlling adaptive gait. Optom. Vis. Sci. 87, 21–27 10.1097/OPX.0b013e3181c1d547 19918210

[B6] Hallemans A. Aerts P. (2009). Effects of visual deprivation on intra-limb coordination during walking in children and adults. Exp. Brain Res. 198, 95–106 10.1007/s00221-009-1937-8 19618172

[B7] Hallemans A. Beccu S. Van Loock K. Ortibus E. Truijen S. Aerts P. (2009a). Visual deprivation leads to gait adaptations that are age- and context-specific: I. Step-time parameters. Gait Posture 30, 55–59 10.1016/j.gaitpost.2009.02.018 19342241

[B8] Hallemans A. Beccu S. Van Loock K. Ortibus E. Truijen S. Aerts P. (2009b). Visual deprivation leads to gait adaptations that are age- and context-specific: II. Kinematic parameters. Gait Posture 30, 307–311 10.1016/j.gaitpost.2009.05.017 19560925

[B9] Hallemans A. Ortibus E. Meire F. Aerts P. (2010). Low vision affects dynamic stability of gait. Gait Posture 32, 547–551 10.1016/j.gaitpost.2010.07.018 20801658

[B10] Iosa M. Fusco A. Morone G. Paolucci S. (2012). Effects of visual deprivation on gait dynamic stability. ScientificWorldJournal 2012:974560 10.1100/2012/974560 22645490 PMC3356761

[B11] Konczak J. (1994). Effects of optic flow on the kinematics of human gait: a comparison of young and older adults. J. Mot. Behav. 26, 225–236 10.1080/00222895.1994.9941678 15757838

[B12] Kushner B. J. (1994). Binocular field expansion in adults after surgery for esotropia. Arch. Ophthalmol. 112, 639–643 10.1001/archopht.1994.01090170083027 8185521

[B13] Legrand A. Quoc E. B. Vacher S. W. Ribot J. Lebas N. Milleret C. (2011). Postural control in children with strabismus: effect of eye surgery. Neurosci. Lett. 501, 96–101 10.1016/j.neulet.2011.06.056 21767607

[B14] Lions C. Bui-Quoc E. Bucci M. P. (2013). Postural control in strabismic children versus non strabismic age-matched children. Graefes Arch. Clin. Exp. Ophthalmol. 251, 2219–2225 10.1007/s00417-013-2372-x 23657730

[B15] Matheron E. Le T. T. Yang Q. Kapoula Z. (2007). Effects of a two-diopter vertical prism on posture. Neurosci. Lett. 423, 236–240 10.1016/j.neulet.2007.07.016 17709195

[B16] Matsuo T. Narita A. Senda M. Hasebe S. Ohtsuki H. (2006). Body sway increases immediately after strabismus surgery. Acta Med. Okayama 60, 13–24 16508685 10.18926/AMO/30754

[B17] Matsuo T. Yabuki A. Hasebe K. Shira Y. H. Imai S. Ohtsuki H. (2010). Postural stability changes during the prism adaptation test in patients with intermittent and constant exotropia. Invest. Ophthalmol. Vis. Sci. 51, 6341–6347 10.1167/iovs.10-5840 20592222

[B18] Odenrick P. Sandstedt P. Lennerstrand G. (1984). Postural sway and gait of children with convergent strabismus. Dev. Med. Child Neurol. 26, 495–499 10.1111/j.1469-8749.1984.tb04477.x 6479470

[B19] Przekoracka-Krawczyk A. Nawrot P. Czaiska M. Michalak K. P. (2014). Impaired body balance control in adults with strabismus. Vision Res. 98, 35–45 10.1016/j.visres.2014.03.008 24680877

[B20] Reynolds R. F. Day B. L. (2005). Visual guidance of the human foot during a step. J. Physiol. 569, 677–684 10.1113/jphysiol.2005.095869 16179363 PMC1464243

[B22] Rosenbaum A. L. (1999). The goal of adult strabismus surgery is not cosmetic. Arch. Ophthalmol. 117:250 10.1001/archopht.117.2.250 10037573

[B23] Schrager M. A. Kelly V. E. Price R. Ferrucci L. Shumway-Cook A. (2008). The effects of age on medio-lateral stability during normal and narrow base walking. Gait Posture 28, 466–471 10.1016/j.gaitpost.2008.02.009 18400500 PMC2583141

[B24] von Noorden G. K. Campos E. (2002). Binocular Vision and Ocular Motility: Theory and Management of Strabismus. St. Louis, MO: Mosby

[B25] Winter D. A. (1979). Biomechanics of Human Movement. New York, NY: Wiley

[B26] Wortham E. Greenwald M. J. (1989). Expanded Binocular peripheral visual fields following surgery for esotropia. J. Pediatr. Ophthalmol. Strabismus 26, 109–112 2723970 10.3928/0191-3913-19890501-04

